# Experimentally evoked same-sex sexual behaviour in pigeons: better to be in a female-female pair than alone

**DOI:** 10.1038/s41598-018-20128-3

**Published:** 2018-01-26

**Authors:** Łukasz Jankowiak, Piotr Tryjanowski, Tomasz Hetmański, Piotr Skórka

**Affiliations:** 1 Department of Vertebrate Zoology and Anthropology, Institute for Research on Biodiversity, University of Szczecin, Wąska 13, PL-71-415 Szczecin, Poland; 2 Institute of Zoology, Poznan University of Life Sciences, Wojska Polskiego 71C, PL 60-625 Poznań, Poland; 3 Department of Zoology, Pomeranian University, Arciszewskiego 22b, 76-200 Słupsk, Poland; 4 Institute of Nature Conservation, Polish Academy of Sciences, al. Mickiewicza 33, 31-120 Kraków, Poland

## Abstract

Same-sex sexual behaviour has been noted among social animals. However, because of the large number of observations necessary, data from controlled experiments are lacking. In this study, we performed experiments to evaluate the effects of male and female removal in colonies of the feral pigeon (*Columba livia* f. *urbana*). After the experimental removal of males, five long-lasting female-female pairs occurred. We found that those pairs could successfully raise offspring in a manner comparable to female-male pairs. Same-sex sexual behaviour and pairing in females is thus a better alternative to postponed breeding or breeding alone without the help of a partner. In contrast, in the case of female-removal experiments, same-sex pairing behaviour occurred in males as a temporary phenomenon with characteristic mutual aggression. Additionally, under a male-biased sex ratio, we observed father-son and father-daughter copulations. To the best of our knowledge, these results are the first obtained under controlled experimental conditions which demonstrate that the sex ratio of a population can shift the social structure and cause cooperative same-sex breeding behaviour to arise in a monogamous species.

## Introduction

There is much evidence of the widespread existence of same-sex sexual behaviour in animal populations^1^. This behaviour has been found in insects and arachnids^2^, fish^3^, amphibians^4^, reptiles^5^, birds^6^ and many mammals^7^. It usually occurs within individuals of the same species, but there have also been records of same-sex pair bonds of individuals belonging to different species^8^. Research attempting to find and understand the ultimate causes of same-sex sexual behaviour has proposed several adaptive explanations^1,9^. The most common explanations include (i) social glue, which is a method to establish a strong social relationship, thereby reducing tension and conflicts^10–12^; (ii) intersexual conflict, when the same-sex interaction establishes dominance in the hierarchy^13^ or reduces the reproductive success of a competitor^14^; (iii) practice, in which a young individual obtains mating experience^15,16^ or gains the ability to improve their territory acquisition^17^; or (iv) alloparenting, in which fluid sexuality of females may be a mechanism to possess allomothering investment from a female not related to the offspring^18,19^. On the other hand, non-adaptive explanations of same-sex sexual behaviour have been proposed, such as (i) mistaken identity, which explains this behaviour as an error due to lack of sex recognition^20^; (ii) heterosexual deprivation when the population density is high and the sex ratio is skewed towards one sex^21–23^; (iii) an evolutionary by-product, when selection acts on traits linked to sexual responsiveness^24^; (iv) maladaptation if the individual is not well adapted to its environment^25^; (v) intoxication, when methylmercury causes same-sex sexual behaviour and diminishes reproductive output as a result^26^; or (vi) nutritional rewards, which is based on evidence that the brain network of the sexual response cycle pathway is similar to the pathway of the pleasure cycle during food acquisition, so that sex preference in later life depends on which sex was a caregiver during infancy^27^.

To date, many correlative studies have analysed same-sex sexual behaviour^6^, but only a small number of experiments have been performed under controlled conditions^20^, especially in the case of vertebrates^26,28^. Controlled removal of one sex was done in a study on the ring-billed gull (*Larus delawarensis*) and the California gull (*L. californicus*)^29^. The results showed that with a female-skewed sex ratio, female-female (hereafter f-f, with male similarly denoted as m) pairs occurred; however, those studies did not observe such behaviour directly and instead, the evidence of f-f pairings was obtained by observation of supernormal clutches. These results could thus be explained by the heterosexual deprivation hypothesis. In another experimental study on the zebra finch (*Taeniopygia guttata*)^30^ where one sex was eliminated, same-sex bonds occurred; the authors explained this behaviour by the social partnership hypothesis, a variation of the alloparenting hypothesis. Moreover, a study of a wild population of the Laysan albatross (*Phoebastria immutabilis*) revealed that f-f mating as a sexual strategy could be adaptive in a female-skewed population^16^. However, we were unable to find an experimental study on any one species that simultaneously tested the adaptive value of same-sex pairing under a skewed sex ratio towards both males and females.

The frequency of m-m sexual behaviour in birds was shown to be lower in monogamous species and higher in more polygamous species^31^. For females, the effect was in the opposite direction since the highest occurrence of f-f pairs occurred in monogamous species and was much lower in species with polygamous reproductive systems^31^. The adaptive value of female-female pairing in monogamous birds may be explained by the alloparenting hypothesis^18,19^, because biparental care is usually required for offspring survival and the availability of an opposite-sex partner is demographically and/or behaviourally limited^29,31^.

Therefore, the main aim of our experimental study was to test the adaptive role of same-sex sexual behaviour in the monogamous feral pigeon (*Columba livia* f. *urbana*). Controlled experimental conditions allowed us to survey both opposite-sex and same-sex sexual behaviour of males and females occurring under a skewed sex ratio in the studied populations. First, we examined whether same-sex sexual behaviour arises by mistake, which implies that same-sex mating should be similarly frequent in both sexes. Next, we asked whether f-f pairing of fertile females produces offspring and compared the performance of f-f pairs to that of single females, which should have a lower chance of achieving breeding success. To test this, we checked the breeding parameters of f-f pairs, f-m pairs and single females in a population with a female-skewed sex ratio. We compared the development of chicks from these three social contexts. Lastly, we investigated the reasons for m-m sexual behaviour by means of two experiments with male-skewed populations.

## Results

### Female-biased experiment

The final pairing structure in the colony after male removal was 20 breeding f-m pairs, five f-f pairs and 14 single females. All f-f pairs had their own territories and nests. The relationships within the f-f pairs were much weaker than within the f-m pairs. Although f-f pairs fought for their territories and moderately defended nests, they also abandoned the nest if a male entered their territory. Female-female pairs had no incentive to defend their territories.

The number of broods differed significantly between the different group statuses (F_2_ = 53.73, p < 0.001). We found significant differences between groups (each comparison p < 0.01) in the mean number of broods (f-m pairs, 5.3 ± 0.22; f-f pairs, 3.8 ± 0.43; single females, 1.86 ± 0.25).

We found that parental status was a significant predictor of incubation time (χ^2^ = 35.92, df = 2, p < 0.001) and that single birds needed more time (21.1 ± 0.26 days) to incubate than f-m pairs (18.9 ± 0.19 days) or f-f pairs (19.2 ± 0.23 days) (both comparisons p < 0.001), but we did not find a significant difference between f-m and f-f pairs (p = 0.769).

In the case of brood overlaps, we did not find significant differences between f-m and f-f pairs (χ^2^ = 0.188, df = 1, p = 0.66). Females that formed f-f pairs were significantly heavier than females that remained alone (396.2 ± 6.72 g *vs*. 375.0 ± 6.72 g, F_2_ = 4.88, p = 0.039).

**Figure 1:**
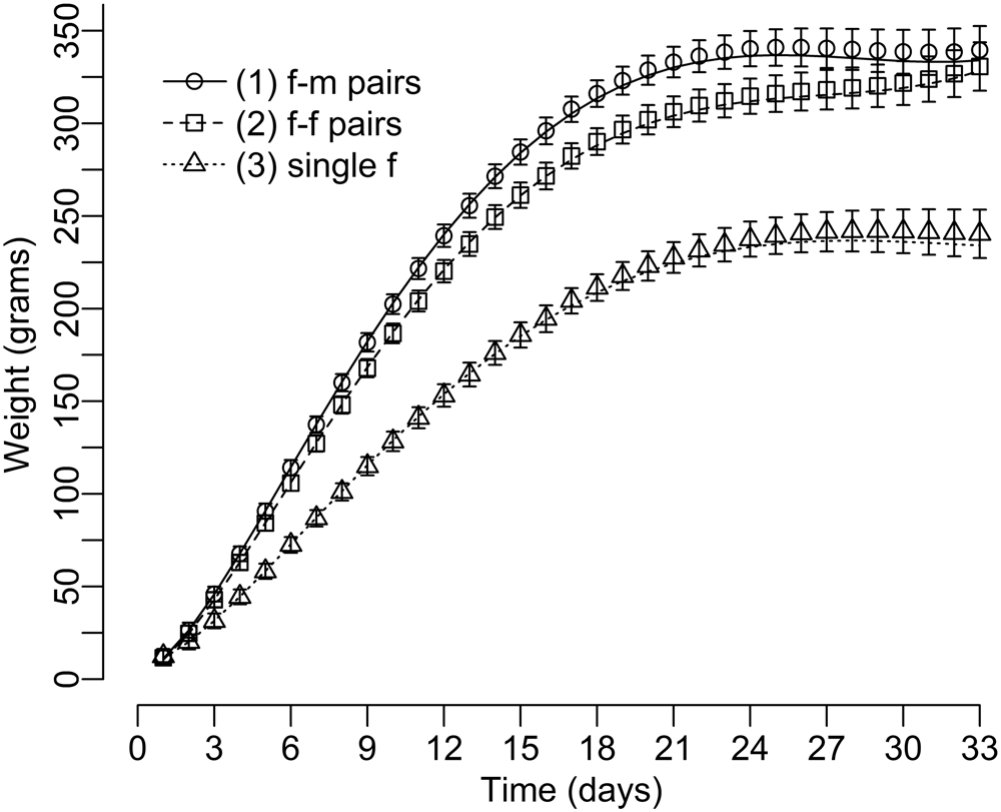
Growth curve showing the body mass gain of chicks raised by pigeons in different pair structures: opposite-sex (female, male: f-m), same-sex (female, female: f-f) and single (only female: f). The values were obtained from the most parsimonious model, and the error bars represent the 95% confidence intervals. The sample size was: 18 hatchling (9 females and 9 males) histories from five same-sex f-f pairs, 36 hatchling histories (19 males and 17 females) from 18 opposite-sex pairs and 14 hatchling histories (6 males and 7 females, 1 chick not sexed) from 14 single breeding females.

The optimal model for chick body mass gain (Table 1, Fig. 1) showed that there was a significantly smaller body mass gain in chicks raised by single females than offspring raised by f-f or f-m pairs (both p < 0.001). However, the difference between f-f and f-m pairs was non-significant (p = 0.198).

**Table 1:** The estimated effects in the most parsimonious model (GLMM with a normal error distribution and two random (r) effects) describing changes in the body mass of pigeon chicks.

Fixed effects:	Estimate	SE	df	t	P
(Intercept)	3.739	3.887	31.7	0.962	0.343
term1	0.062	0.002	1719.0	31.507	<0.001
status = f-f	−0.147	6.563	37.5	−0.022	0.982
status = f	3.390	7.139	43.2	0.475	0.637
term2	7.364	0.148	351.7	49.770	<0.001
term3	−2.600	0.058	696.1	−45.159	<0.001
term1: status = f-f	−0.001	0.003	1722.0	−0.328	0.743
term1: status = f	−0.032	0.004	1762.0	−8.271	<0.001
status = f-f × term2	−0.480	0.254	353.2	−1.888	0.060
status = f × term2	−3.178	0.284	378.9	−11.175	<0.001
status = f-f × term3	0.145	0.099	699.3	1.470	0.142
status = f × term3	1.182	0.112	752.1	10.581	<0.001
hatchling id (r)	233.028	15.265			
time/hatchling id (r)	4.857	2.204			
nest id (r)	111.869	10.577			

The time structure was obtained by a fractional polynomial technique and the time effects are denoted as follows: term1 = time^3^, term2 = time^2^, term3 = time^2^ × log(time). f-f = female-female pair, f = single female.

We found significant differences in the number of hatchlings per brood based on group status (χ^2^ = 17.775, df = 2, p < 0.001); single females had significantly fewer hatchlings (0.69 ± 0.16) than f-m (1.71 ± 0.13, p < 0.001) but not significantly fewer hatchlings than f-f pairs (1.26 ± 0.26, p = 0.130). We did not find a significant difference between f-m and f-f pairs (p = 0.347).

We found significant differences in the number of fledglings per brood based on group status (χ^2^ = 29.783, df = 2, p < 0.001); single females had significantly fewer fledglings (0.42 ± 0.13) than f-m (1.58 ± 0.12, p < 0.001) but not significantly fewer than f-f pairs (0.89 ± 0.22, p = 0.129). The difference between f-m and f-f pairs was almost significant (p = 0.064).

### First male-biased sex ratio experiment

In the experimental colony where there were twice as many males as females (20 females and 45 males), 20 f-m pairs emerged and 25 males remained alone. The single sexually mature males included two-year-old birds without reproductive experience (n = 10) as well as 3–4-year-old males with reproductive experience (n = 15). During the experiment, only three polyandrous groups occurred where males joined a f-m pair. The duration of these groups was very short-term, and these groups always consisted of one female paired with the dominant male that tolerated a subordinate male in his territory. Both males competed for copulation with the female and for access to the nest. The subordinate male had access to the nest and could incubate eggs or feed the chicks but only during the absence of the alpha male. Such polyandrous groups lasted for only 1–2 broods. Of the 25 single males, only four created two m-m pairs. In those pairs, we observed same-sex pairing behaviour as i) five courtships and attempts at “kissing” and ii) four short-term pairings involving mating in the nest with copulation and the establishment of a nest territory with the same-sex individual with courtships. Within the m-m pair, we did not observe nest building or adoption of planted eggs. The m-m pairs were unstable and lasted only a few weeks, during which same-sex sexual behaviour and aggressive behaviour between males were observed.

### Second male-biased sex ratio experiment

The widowing experiment revealed that after females had been removed, males displayed mating behaviour towards their offspring. Such behaviour was observed in five of the 15 experimentally widowed males. Chicks came back to their father’s territory and were fed by him. After feeding, the male started courtship with the chick that often resulted in copulation. Of the five pairs, four pairs were father-son and one was father-daughter. Father-son pairs were persistent for the period of growth of the latter. In addition to copulation, we observed mate guarding, which is characteristic of males watching fertile females before egg laying. The relationship between father and son weakened when the son reached sexual maturity. We did not observe any nest building or adoption of experimentally provided eggs by father-son pairs.

## Discussion

Our results indicate that alloparenting and heterosexual deprivation are possible reasons for the occurrence of same-sex sexual behaviour in females and males, respectively. This is also the first described case of same-sex bonding in pigeons. We found that f-f pairs could successfully raise offspring, in contrast to m-m pairs, which were of short duration and in which we did not observe adoption of planted eggs. This result is in agreement with our prediction that female same-sex bonds often occur in monogamous birds^31^. The heterosexual deprivation hypothesis^21–23^ explains same-sex sexual behaviour as an effect of the unavailability of an opposite-sex partner. On the other hand, we found that this behaviour may have an adaptive function in f-f pairs, as predicted by the alloparenting hypothesis^18,19^. The development of chicks, incubation time, overlap between broods, and numbers of hatchlings and fledglings of f-f pairs did not differ significantly from f-m pairs, but the number of broods did and was higher for f-m pairs. The number of potential mates can affect the choice of reproductive strategy: in general, the lower the density of males, the less selective the females^32^. Thus, f-f pairing could have evolved as a different mating strategy under a highly female-skewed sex ratio. The adaptive value of f-f pairing could be regarded as “making the best of a bad job”^9^ under a female-skewed sex ratio because it is better to form a same-sex pair and breed than to breed alone or not to breed at all. Since f-f pairs are rarely found in populations with equal sex ratios, this behaviour should be considered in terms of both alloparenting and heterosexual deprivation hypotheses.

Under a skewed sex ratio within social groups, subordinate individuals have a limited chance of reproducing with an opposite-sex partner and there is a higher probability of same-sex sexual behaviour^33^. This behaviour could also arise as a dominance-related or affiliative socio-sexual function for dominant or subordinate individuals within social groups^6^. However, our observation that f-f pairs are formed by females that are heavier than single females does not support this hypothesis. Similar findings were revealed in surveys of human sexuality, in which non-heterosexual women have a larger body weight than heterosexual women^34^. Body size and condition are thought to be the best signals of fighting or competitive abilities because size and condition often reflect individual strength^35–38^. This may be crucial during the formation of same-sex pair bonds since females may prefer larger same-sex mates in order to achieve greater breeding success. As pigeons forage in flocks, competition for food among individuals may be substantial^39^. It has been shown that dominance in feeding areas may be directly linked with breeding success and offspring growth rate^40^. Thus, mating with heavier females may compensate for the lack of a male.

In our study, m-m pairings were much less frequent and required very special conditions to arise (e.g. widowed males). The behaviour between males within m-m pairs was very aggressive and they quickly broke up. This confirms the view that same-sex bonds rarely emerge and are sustained in male monogamous birds^31^. In this experiment, m-m pairs did exist but as a temporary phenomenon characterised by mutual aggression. There was no adoption of planted eggs in m-m pairs. It should be noted, however, that the interpretation of this behaviour is ambiguous and could be alternatively explained as mating error. Thus, it seems that male same-sex sexual behaviour is mainly explained by the heterosexual deprivation hypothesis as a result of the “prison effect”, when the sex ratio is skewed strongly towards males. It has been shown that a male-biased sex ratio in bird populations is related to egg damage caused by unpaired males^41^. This behaviour can be considered a form of infanticide. Furthermore, this act influences divorce of females and increases the chances of the unpaired male bird pairing with the divorced female.

A meta-analysis of same-sex sexual behaviours in birds with different reproductive strategies^6^ showed that in species where the male provides less parental care than the female, the frequency of m-m pairing increases, but if the male provides more parental care than the female, then there is a smaller chance of m-m pairings. Our results from a male-skewed sex ratio experiment confirmed this relationship by showing that the level of parental care provided by male pigeons is equivalent to that provided by females and relatively higher than in polygamous species. Thus, we should not have expected to observe same-sex pairings of males, and our experiment confirmed that m-m pairs were not stable. The above-mentioned meta-analysis^6^ also revealed that female same-sex sexual behaviour in socially monogamous species often results in f-f pairings. If females of monogamous species spend more time than males caring for siblings, they show a higher rate of same-sex sexual behaviours, such as pair bonding and courtship. This explains the very rare occurrence of female same-sex sexual behaviour in polygynous systems. In such sexual systems, females invest much more in broods than males compared to monogamous species^6^.

The evoked f-f pair bonding in our study is the result of two factors: a monogamous system and an experimentally skewed sex ratio. Two hypotheses seem to contribute to the explanation of same-sex sexual behaviour in the species studied: alloparenting in females and heterosexual deprivation in males. If the male partner of a female deserts the brood, divests paternal resources due to extra-pair mating effort or dies, female same-sex sexual behaviour and pairing may have an adaptive value because pairing with another female can act as compensation for breeding failure. Additionally, the females searching for a heavy female mate could be due to the anticipated provision of sufficient food. Nevertheless, because this behaviour occurs in sex-biased conditions, f-f pairing may also occur during a shortage of males in the population. Notably, heterosexual deprivation seems to be especially plausible for explaining the occurrence of m-m pairs because they were short-lasting and did not result in breeding success when eggs were experimentally planted.

## Methods

### Artificial colonies

The study was carried out in 2007 and 2009 in artificially established colonies of the feral pigeon in a building in NW Poland, to which individuals were transported from four feral pigeon breeding colonies in Słupsk (NW Poland, 54° 28′N, 17° 10′E) in 2005. Only fledglings formed the artificial colonies, which were completed over several months each time. The overall number of introduced birds in all experiments was 116. All pigeons were individually colour-ringed, and to reduce the variability in breeding performance associated with age^42^, all birds used in the experiments were at least 2 years old.

We noted the dates of same-sex pair formation and their persistence during the breeding period. All observations of bird behaviour were performed from a hide and lasted 3–7 hours per day. The sex of the pigeons was determined according to the characteristic mating behaviours of the sexes at an older age^43^. These sexual behaviours are highly sex-specific, e.g. only males bow-coo, females always initiate heteropreening, courtship feeding, driving and female guarding are always performed by a male^43^. Also, copulation is initiated by a male mounting a female^43^. Every individual was reliably sexed according to these types of behaviour. We considered all attempts at “kissing”, copulation and establishing a nest territory during which mating behaviour towards a same-sex individual was observed as same-sex sexual behaviour of males and females. We did not observe any atypical behaviour of females in f-f pairs. Females from f-f pairs behaved in the same way as females from f-m pairs. However, females from f-f pairs copulated with other males (in this way eggs were fertilised). There was also one clear difference between f-f and f-m pairs: in f-f pairs there was no driving/mate guarding because no male was present. The experimental removal of males did not change the “female-like” mating behaviour of females.

The major breeding strategy of the feral pigeon is to hatch as many broods as possible. Each brood always consists of two eggs, so increasing the number of broods in one breeding season is an adaption to increase breeding output^44,45^. As chicks become normothermic, the adults begin to prepare for the next brood. The overlapping broods require the cooperation of two parents; for 80% of the time, the new clutch is incubated by females, while males feed the fledglings from the previous brood. After the new brood hatches, both parents feed their offspring.

### Experiment creating a female-biased sex ratio

The experiment was conducted between February 2007 and February 2009. In February 2007, we removed 18 paired males and all young unpaired males (the offspring from 2006) from the first artificial colony. The removed individuals were housed in a separate room and given water and food *ad libitum*.

At the end, the structure of the colony was 20 f-m pairs (total 106 broods), five f-f pairs (total 19 broods) and 14 single females (total 26 broods; note that for single breeding females, we added two pairs [two broods - four hatchling histories] from 2009 in which the experimental conditions were the same as described above). The hatching success (measured as the number of hatched eggs) and fledglings per brood was obtained for 106 f-m broods, 19 f-f broods and 26 single females’ broods. For further analyses we selected the histories of 18 broods (one brood per pair; two broods from two pairs were excluded because the relevant data became lost) from 20 f-m pairs. Thus, the sample sizes for the incubation time were 18 f-m broods, 13 broods of f-f pairs (six broods excluded because females abandoned their nest) and 12 single females’ broods (14 broods excluded because females abandoned their nests). In the case of sample sizes for brood overlap (the number of days from the hatching of chicks to the next egg laying event) we had 18 f-m broods and 13 f-f broods (six broods excluded because females abandoned their nests); single females’ broods were not analysed because females laid eggs only 3 times while they were feeding their previous brood.

To obtain the mass gain curve of hatchlings, we analysed only the successfully hatching broods. Hence, we analysed 18 hatchling (9 females and 9 males) histories from five same-sex f-f pairs, 36 hatchling histories (19 males and 17 females) from 18 opposite-sex pairs and 14 hatchling histories (6 males and 7 females, 1 chick not sexed) from 14 single breeding females. The sex distribution of offspring was evenly spread across the groups (χ^2^ = 0.173, df = 2, p = 0.917). The masses of the nestlings were recorded every day from hatching until fledging (33 days) using a PESOLA spring scale. Opposite-sex pigeon pairs always have two eggs, so if females in f-f pairs laid more than two eggs, we randomly removed excess eggs from the nest; this was the case for 17 broods. The removal of excess eggs was necessary because pigeons can only raise two chicks^43^. Before the experiment started we had measured the weight of females without any knowledge of their future pair associations; thus, during the experiment we had 10 females in f-f pairs and 10 single females to test whether the larger weight of females is associated with f-f pairing.

Breeding parameters such as the number of broods per season and the overlap between broods (days) were analysed using general linear models. For incubation time (days), we used a general linear mixed model, and for the number of hatchlings and fledglings per brood, we used generalised linear mixed models with a Poisson error distribution. For mixed models, we set the identity of the pair (id) as a random effect. The weight of single females against f-f paired females was tested using a general linear model. All statistics were performed using the R statistical software^46^ and the additional “lme4” package^47^. The explanatory variables were tested using the drop1() function. The assumptions of all models were checked graphically, but we did not find any violation of either homoscedasticity or normality of residuals.

To fit the model to mass gain of pigeon chicks, we searched for the best structure of the mass growth curve. To obtain the most parsimonious time structure we used fractional polynomials^48^. This method is based on the fit of regression models that have *m* terms of the form t^*p*^, where *p* is defined as the exponents selected from the small set integer and non-integer values (S = {−2, −1, −0.5, 0, 0.5, 1, 2, 3}). The linear predictor is defined by

$${\beta }_{0}+\sum _{m=1}^{M}{\beta }_{m}{t}^{{p}_{m}}$$

where t is a time covariate (days) and *M* is the order of covariate t. For each term, the power *p*_*m*_ is selected from the restricted set S. Note that according to^48^, when *M* = 2 and p_1_ = p_2_, the linear predictor (1) is β_0_ + β_1_t^p1^ + β_2_t^p1^ log(t) and when *p* = 0, the predictor is log (t). We fitted all possible models from M = 1 to M = 3. All performed models are presented in Appendix 1. The “best” model was chosen using Akaike’s Information Criterion (AIC, the lower the value, the better fitted the model). This procedure penalises the addition of new parameters, so it helps to avoid over-fitting^49^. The computational process of fractional polynomials was done using the package “CorrMixed”^50^ in the R program^46^.

Subsequently, the selected structure of the time covariate was used as a covariate in a GLMM (general linear mixed model with normal error structure). To compare different parent structures, we allowed this factor (three levels: f-m, f-f, and single) to interact with the polynomial time covariate. We used a mixed model because the measurements of chicks were sequential data taken every day until fledging (33 days). We checked for different random effect structures. In the most general structure, we added hatchling id, pair id or nest id and the time of measurement as random effects. We found the random slope of time for each pair (time/id) and the random intercept of nest id with the lowest AIC value. We verified the model graphically to check for model assumptions and did not find any violations.

The parameter estimations and estimated means are given with standard errors (SE). Multiple comparisons were carried out using Tukey’s test.

### First experiment creating a male-biased sex ratio

In 2008, we established the second artificial colony. At the beginning of the breeding season (in February) we removed 20 sexually mature paired females (with and without breeding experience). The removed individuals were housed in a separate room and were given water and food *ad libitum*. The male-biased colony structure consisted of 20 opposite-sex pairs and 25 single unpaired males. In this experiment, we created conditions for egg adoption. If two males were observed in a nest we added one egg and observed the outcome, i.e. whether the egg was adopted and incubated or abandoned.

### Second experiment creating a male-biased sex ratio

The experiment was planned after field observations which revealed that single males may show mating behaviour towards fledglings. The experiment was performed on the third artificial colony in 2008. We widowed 15 males (by removing the females and placing them in another room) just before the chicks fledged. This was performed under conditions of a male-skewed sex ratio. To facilitate the father-son pairs, the lighter of the two orphaned chicks was removed. Earlier studies showed that young males are heavier than young females^42,51^; these removed chicks must have been females. There are significant differences between sexes in postembryonal development; after the fifth day of life males become heavier than females. This difference increases in time and males are 25 grams heavier than females on average before fledgling. In broods consisting of two female offspring, this method may not be reliable. However, as the young birds had been ringed, the final identification of sex was confirmed as the birds grew up, based on their adult sex-specific behaviour^52^.

### Data availability

The authors declare that data supporting the findings of this study are available within the paper and the supplementary information files.

### Ethical note

This study complied with Polish regulations regarding the ethical treatment of research subjects in the experimental period 2007–2010, and the colony was established with the approval of the relevant authorities, the University Dean (“Zezwolenie Dziekana Wydziału Mat-Przyr Akademii Pomorskiej na prowadzenie badań na gołębiu miejskim 2/2010”) and with approval of the Ministry of the Environment (“Zmienność parametrów hematologicznych i biochemicznych u gołębia miejskiego (*Columba livia f. urbana*) w różnych okresach lęgu”, approval 20.07.2007, decision number: DLOPiK - op/ogiz-4200/III-21/3706/07/jr).

## Electronic supplementary material

Supplementary information

Supplementary Dataset

## References

[1] Bailey NW, Zuk M (2009). Same-sex sexual behavior and evolution. Trends Ecol. Evol..

[2] Scharf I, Martin OY (2013). Same-sex sexual behavior in insects and arachnids: Prevalence, causes, and consequences. Behav. Ecol. Sociobiol..

[3] Banerjee SB, Adkins-Regan E (2014). Same-sex partner preference in adult male zebra finch offspring raised in the absence of maternal care. Anim. Behav..

[4] Marco A, Lizana M (2002). The absence of species and sex recognition during mate search by male common toads, Bufo bufo. Ethol. Ecol. Evol..

[5] Rodrigues JFM, Liu Y, Werner YL (2016). Revisiting the same-sex mounting in chelonians under the concept of whole-animal. J. Ethol..

[6] MacFarlane GR, Blomberg SP, Vasey PL (2010). Homosexual behaviour in birds: Frequency of expression is related to parental care disparity between the sexes. Anim. Behav..

[7] Fruth, B. & Hohmann, G. Social grease for females? Same-sex genital contacts in wild bonobos. In *Homosexual Behaviour in Animals* (eds Sommer, V. & Vasey, P. L.) 294–315 (Cambridge University Press, 2006).

[8] Betleja J, Skórka P, Zielinska M (2007). Super-normal Clutches and Female-female Pairs in Gulls and Terns Breeding in Poland. Waterbirds.

[9] Poiani, A. *Animal homosexuality: A biosocial perspective*. (Cambridge University Press, 2010).

[10] Mann, J. Establishing trust: socio-sexual behaviour and the development of male-male bonds among Indian Ocean bottlenose dolphins. In *Homosexual Behaviour in Animals* (eds Sommer, V. & Vasey, P. L.) 107–130 (Cambridge University Press, 2006).

[11] MacRoberts, M. H. & MacRoberts, B. R. *Social Organization and Behavior of the Acorn Woodpecker in Central Coastal California (Ornithology Monographs*, *Vol. 21)*. (American Ornithologists’ Union, 1976).

[12] Vasey PL, Bernard C, Gauthier C (1998). Mounting interaction between female Japanese macaques: Testing the influence of dominance & aggression. Ethology.

[13] Vervaecke, H. & Roden, C. Going with the herd: same-sex interaction and competition in American bison. In *Homosexual Behaviour in Animals* (eds Sommer, V. & Vasey, P. L.) 131–153 (Cambridge University Press, 2006).

[14] Preston-Mafham K (2006). Post-mounting courtship and the neutralizing of male competitors through “homosexual” mountings in the fly Hydromyza livens F. (Diptera: Scatophagidae). J. Nat. Hist..

[15] McRobert SP, Tompkins L (1988). Two Consequences of Homosexual Courtship Performed by Drosophila melanogaster and Drosophila affinis Males. Evolution (N. Y)..

[16] Young LC, VanderWerf EA (2014). Adaptive value of same-sex pairing in Laysan albatross. Proc. Biol. Sci..

[17] King, C. E. Pink flamingos: atypical partnerships and sexual activity in colonially breeding birds. In *Homosexual Behaviour in Animals* (eds Sommer, V. & Vasey, P. L.) 77–106 (Cambridge University Press, 2006).

[18] Kuhle BX, Radtke S (2013). Born both ways: The alloparenting hypothesis for sexual fluidity in women. Evol. Psychol..

[19] Kuhle, B. X. & Brezinski, S. Alloparenting and Female Same-Sex Behavior. In *Encyclopedia of Evolutionary Psychological Science* (eds Weekes-Shackelford, V. A. & Shackelford, T. K.) 1–5 (Springer International Publishing, 2016).

[20] Engel KC, Männer L, Ayasse M, Steiger S (2015). Acceptance threshold theory can explain occurrence of homosexual behaviour. Biol. Lett..

[21] Bonnet X (2016). A prison effect in a wild population: a scarcity of females induces homosexual behaviors in males. Behav. Ecol..

[22] Leca JB, Gunst N, Vasey PL (2014). Male homosexual behavior in a free-ranging all-male group of Japanese macaques at Minoo, Japan. Arch. Sex. Behav..

[23] Vasey PL, Gauthier C (2000). Skewed sex ratios and female homosexual activity in Japanese macaques: An experimental analysis. Primates.

[24] Ottenheimer-Carrier L, Leca JB, Pellis S, Vasey PL (2015). A structural comparison of female-male and female-female mounting in Japanese macaques (Macaca fuscata). Behav. Processes.

[25] Bagemihl, B. *Biological Exuberance*. (St. Martin’s Press, 1999).

[26] Frederick P, Jayasena N (2011). Altered pairing behaviour and reproductive success in white ibises exposed to environmentally relevant concentrations of methylmercury. Proc. Biol. Sci..

[27] Luoto S, Rantala MJ (2017). Specificity of Women’s Sexual Response: Proximate Mechanisms and Ultimate Causes. Arch. Sex. Behav..

[28] Bierbach D, Jung CT, Hornung S, Streit B, Plath M (2012). Homosexual behaviour increases male attractiveness to females. Biol. Lett..

[29] Conover MR, Hunt GL (1981). Experimental Evidence That Female-Female Pairs in Gulls Result From a Shortage of Breeding Males. Condor.

[30] Elie JE, Mathevon N, Vignal C (2011). Same-sex pair-bonds are equivalent to male-female bonds in a life-long socially monogamous songbird. Behav. Ecol. Sociobiol..

[31] MacFarlane GR, Blomberg SP, Kaplan G, Rogers LJ (2007). Same-sex sexual behavior in birds: Expression is related to social mating system and state of development at hatching. Behav. Ecol..

[32] Kokko H, Rankin DJ (2006). Lonely hearts or sex in the city? Density-dependent effects in mating systems. Philos. Trans. R. Soc. Lond. B. Biol. Sci..

[33] Poiani A (2008). Same-Sex Mounting in Birds: Comparative Test of a Synthetic Reproductive Skew Model of Homosexuality. Open Ornithol. J..

[34] Boehmer U, Bowen DJ, Bauer GR (2007). Overweight and obesity in sexual minority women: evidence from population-based data. Am. J. Public Health.

[35] Alatalo RV, Moreno J (1987). Body size, interspecific interactions, and use of foraging sites in tits (Paridae). Ecology.

[36] Breitburg DL (1987). Interspecific competition and the abundance of nest sites: factors affecting sexual selection. Ecology.

[37] Lindstrom K (1988). Male-male competition for nest sites in the sand goby, Pomatoschistus minutus. Oikos.

[38] Jonart LM, Hill GE, Badyaev AV (2007). Fighting ability and motivation: determinants of dominance and contest strategies in females of a passerine bird. Anim. Behav..

[39] Przybylska K (2012). Local and landscape-level factors affecting the density and distribution of the Feral Pigeon Columba livia var. domestica in an urban environment. Acta Ornithol..

[40] Hahn S, Bauer S (2008). Dominance in feeding territories relates to foraging success and offspring growth in brown skuas Catharacta antarctica lonnbergi. Behav. Ecol. Sociobiol..

[41] Marchesan M (2002). Operational sex ratio and breeding strategies in the Feral Pigeon Columba livia. Ardea.

[42] Hetmański T, Barkowska M (2008). Breeding Parameters and Recruitment in Feral Pigeons. Acta Ornithol..

[43] Janiga M (1987). Seasonal aspects of intensity and course of daily translocations of pigeons (Columba livia f. domestica) for food from Bratislava to its surroundings. Acta Fac. Rerum nat. Univ. Comenianae. Zool..

[44] Hetmański T (2004). Timing of breeding in the Feral Pigeon Columba livia f. domestica in Slupsk (NW Poland). Acta Ornithol..

[45] Hetmański T, Wołk E (2005). The effect of environmental factors and nesting conditions on clutch overlap in the Feral Pigeon Columba livia f. urbana (Gm.). Polish J. Ecol..

[46] R Core Team. A language and environment for statistical computing, at http://www.r-project.org/ (2014).

[47] Bates, D., Maechler, M., Bolker, B. & Walker, S. lme4: Linear mixed-effects models using Eigen and S4. R package version 1.1–7, http://CRAN.R-project.org/package=lme4. (2014).

[48] Van der Elst W, Molenberghs G, Hilgers RD, Verbeke G, Heussen N (2016). Estimating the reliability of repeatedly measured endpoints based on linear mixed-effects models. A tutorial. Pharm. Stat..

[49] Burnham, K. P. & Anderson, D. R. *Model selection and multimodel inference: a practical information-theoretic approach*. (Springer, 2002).

[50] Van der Elst, W., Molenberghs, G., Hilgers, R. D., Verbeke, G. & Heussen, N. CorrMixed: Estimate Correlations Between Repeatedly Measured Endpoints (E.g., Reliability) Based on Linear Mixed-Effects Models. R package version 0.1–13, at https://cran.r-project.org/package=CorrMixed (2016). 10.1002/pst.1787 27681820

[51] Hetmański, T. *Różnice morfologiczne i behawioralne gołębia miejskiego*. (Wydawnictwo Naukowe Akademii Pomorskiej, 2011).

[52] Johnston, R. F. & Janiga, M. *Feral pigeons*. (Oxford University Press, 1995).

