# Supplementary material for: Experimentally evoked same-sex sexual behaviour in pigeons: better to be in a female-female pair than alone
Source: Sci Rep. 2018 Jan 26;8:1654. doi: 10.1038/s41598-018-20128-3 (PMC5785962; doi:10.1038/s41598-018-20128-3)
Supplement: Supplementary file 1 — Supplementary information [file 41598_2018_20128_MOESM1_ESM.doc]

**Experimentally evoked same-sex sexual behaviour in pigeons: better to be in a female-female pair than alone**

Łukasz JANKOWIAK^a*^, Piotr TRYJANOWSKI^b^ , Tomasz HETMAŃSKI ^c^, & Piotr SKÓRKA^d^

^a^ Department of Vertebrate Zoology and Anthropology, Institute for Research on Biodiversity, University of Szczecin, Wąska 13, PL-71-415 Szczecin, Poland, email: jankowiakl@gmail.com

^b^ Institute of Zoology, Poznan University of Life Sciences, Wojska Polskiego 71 C, PL 60-625 Poznań; Poland; email: piotr.tryjanowski@gmail.com

^c^ Department of Zoology, Pomeranian University, Arciszewskiego 22b, 76-200 Słupsk, Poland; email: tomasz.hetmanski@apsl.edu.pl

^d^ Institute of Nature Conservation, Polish Academy of Sciences, al. Mickiewicza 33, 31-120 Kraków, Poland, email: skorasp@poczta.onet.pl

Table. All performed models to obtain the most parsimonious non-linear time structure using fractional polynomials to obtain the chicks’ mass growth curve. Power1, power2, power 3 are defined as exponent of the growth curve. AIC – Akaike information criterion.

| power1 | power2 | power3 | AIC |
| --- | --- | --- | --- |
| 2 | 2 | 3 | 16290.85 |
| 3 | 2 | 2 | 16290.85 |
| 0.5 | 0.5 | 1 | 16298.22 |
| 1 | 0.5 | 0.5 | 16298.22 |
| 0 | 1 | 1 | 16298.98 |
| 1 | 1 | 0 | 16298.98 |
| 0.5 | 1 | 1 | 16300.92 |
| 1 | 1 | 0.5 | 16300.92 |
| 2 | 3 | 3 | 16302.4 |
| 3 | 3 | 2 | 16302.4 |
| -0.5 | 1 | 1 | 16303.09 |
| 1 | 1 | -0.5 | 16303.09 |
| -0.5 | 0.5 | 2 | 16304.82 |
| -0.5 | 2 | 0.5 | 16304.82 |
| 0.5 | -0.5 | 2 | 16304.82 |
| 0.5 | 2 | -0.5 | 16304.82 |
| 2 | -0.5 | 0.5 | 16304.82 |
| 2 | 0.5 | -0.5 | 16304.82 |
| 0 | 0.5 | 2 | 16305.11 |
| 0 | 2 | 0.5 | 16305.11 |
| 0.5 | 0 | 2 | 16305.11 |
| 0.5 | 2 | 0 | 16305.11 |
| 2 | 0 | 0.5 | 16305.11 |
| 2 | 0.5 | 0 | 16305.11 |
| 0 | 0.5 | 1 | 16305.94 |
| 0 | 1 | 0.5 | 16305.94 |
| 0.5 | 0 | 1 | 16305.94 |
| 0.5 | 1 | 0 | 16305.94 |
| 1 | 0 | 0.5 | 16305.94 |
| 1 | 0.5 | 0 | 16305.94 |
| -1 | 0.5 | 2 | 16311.54 |
| -1 | 2 | 0.5 | 16311.54 |
| 0.5 | -1 | 2 | 16311.54 |
| 0.5 | 2 | -1 | 16311.54 |
| 2 | -1 | 0.5 | 16311.54 |
| 2 | 0.5 | -1 | 16311.54 |
| -1 | 1 | 1 | 16311.84 |
| 1 | 1 | -1 | 16311.84 |
| 0.5 | 0.5 | 2 | 16313.19 |
| 2 | 0.5 | 0.5 | 16313.19 |
| -2 | 1 | 2 | 16315.76 |
| -2 | 2 | 1 | 16315.76 |
| 1 | -2 | 2 | 16315.76 |
| 1 | 2 | -2 | 16315.76 |
| 2 | -2 | 1 | 16315.76 |
| 2 | 1 | -2 | 16315.76 |
| -1 | 1 | 2 | 16317.35 |
| -1 | 2 | 1 | 16317.35 |
| 1 | -1 | 2 | 16317.35 |
| 1 | 2 | -1 | 16317.35 |
| 2 | -1 | 1 | 16317.35 |
| 2 | 1 | -1 | 16317.35 |
| 1 | 3 | 3 | 16318.42 |
| 3 | 3 | 1 | 16318.42 |
| -0.5 | 0 | 3 | 16319.21 |
| -0.5 | 3 | 0 | 16319.21 |
| 0 | -0.5 | 3 | 16319.21 |
| 0 | 3 | -0.5 | 16319.21 |
| 3 | -0.5 | 0 | 16319.21 |
| 3 | 0 | -0.5 | 16319.21 |
| -1 | 0.5 | 3 | 16319.75 |
| -1 | 3 | 0.5 | 16319.75 |
| 0.5 | -1 | 3 | 16319.75 |
| 0.5 | 3 | -1 | 16319.75 |
| 3 | -1 | 0.5 | 16319.75 |
| 3 | 0.5 | -1 | 16319.75 |
| -0.5 | 1 | 2 | 16319.84 |
| -0.5 | 2 | 1 | 16319.84 |
| 1 | -0.5 | 2 | 16319.84 |
| 1 | 2 | -0.5 | 16319.84 |
| 2 | -0.5 | 1 | 16319.84 |
| 2 | 1 | -0.5 | 16319.84 |
| 0 | 1 | 2 | 16323.29 |
| 0 | 2 | 1 | 16323.29 |
| 1 | 0 | 2 | 16323.29 |
| 1 | 2 | 0 | 16323.29 |
| 2 | 0 | 1 | 16323.29 |
| 2 | 1 | 0 | 16323.29 |
| -0.5 | 0.5 | 3 | 16323.31 |
| -0.5 | 3 | 0.5 | 16323.31 |
| 0.5 | -0.5 | 3 | 16323.31 |
| 0.5 | 3 | -0.5 | 16323.31 |
| 3 | -0.5 | 0.5 | 16323.31 |
| 3 | 0.5 | -0.5 | 16323.31 |
| -0.5 | 0.5 | 1 | 16323.56 |
| -0.5 | 1 | 0.5 | 16323.56 |
| 0.5 | -0.5 | 1 | 16323.56 |
| 0.5 | 1 | -0.5 | 16323.56 |
| 1 | -0.5 | 0.5 | 16323.56 |
| 1 | 0.5 | -0.5 | 16323.56 |
| 0 | 0.5 | 0.5 | 16324.41 |
| 0.5 | 0.5 | 0 | 16324.41 |
| -2 | 0.5 | 3 | 16324.84 |
| -2 | 3 | 0.5 | 16324.84 |
| 0.5 | -2 | 3 | 16324.84 |
| 0.5 | 3 | -2 | 16324.84 |
| 3 | -2 | 0.5 | 16324.84 |
| 3 | 0.5 | -2 | 16324.84 |
| 0.5 | 1 | 2 | 16326.85 |
| 0.5 | 2 | 1 | 16326.85 |
| 1 | 0.5 | 2 | 16326.85 |
| 1 | 2 | 0.5 | 16326.85 |
| 2 | 0.5 | 1 | 16326.85 |
| 2 | 1 | 0.5 | 16326.85 |
| -0.5 | 0 | 2 | 16329.3 |
| -0.5 | 2 | 0 | 16329.3 |
| 0 | -0.5 | 2 | 16329.3 |
| 0 | 2 | -0.5 | 16329.3 |
| 2 | -0.5 | 0 | 16329.3 |
| 2 | 0 | -0.5 | 16329.3 |
| 1 | 1 | 2 | 16329.68 |
| 2 | 1 | 1 | 16329.68 |
| 1 | 2 |  | 16330.17 |
| 2 | 1 |  | 16330.17 |
| 1 | 2 | 3 | 16331.61 |
| 1 | 3 | 2 | 16331.61 |
| 2 | 1 | 3 | 16331.61 |
| 2 | 3 | 1 | 16331.61 |
| 3 | 1 | 2 | 16331.61 |
| 3 | 2 | 1 | 16331.61 |
| 1 | 2 | 2 | 16332.11 |
| 2 | 2 | 1 | 16332.11 |
| -2 | 1 | 1 | 16332.44 |
| 1 | 1 | -2 | 16332.44 |
| 0 | 0.5 | 3 | 16332.76 |
| 0 | 3 | 0.5 | 16332.76 |
| 0.5 | 0 | 3 | 16332.76 |
| 0.5 | 3 | 0 | 16332.76 |
| 3 | 0 | 0.5 | 16332.76 |
| 3 | 0.5 | 0 | 16332.76 |
| -2 | 0.5 | 2 | 16333.01 |
| -2 | 2 | 0.5 | 16333.01 |
| 0.5 | -2 | 2 | 16333.01 |
| 0.5 | 2 | -2 | 16333.01 |
| 2 | -2 | 0.5 | 16333.01 |
| 2 | 0.5 | -2 | 16333.01 |
| -1 | 0 | 3 | 16346.07 |
| -1 | 3 | 0 | 16346.07 |
| 0 | -1 | 3 | 16346.07 |
| 0 | 3 | -1 | 16346.07 |
| 3 | -1 | 0 | 16346.07 |
| 3 | 0 | -1 | 16346.07 |
| 0.5 | 0.5 | 3 | 16346.47 |
| 3 | 0.5 | 0.5 | 16346.47 |
| -1 | 0.5 | 1 | 16347.06 |
| -1 | 1 | 0.5 | 16347.06 |
| 0.5 | -1 | 1 | 16347.06 |
| 0.5 | 1 | -1 | 16347.06 |
| 1 | -1 | 0.5 | 16347.06 |
| 1 | 0.5 | -1 | 16347.06 |
| 1 | 1 | 3 | 16350.86 |
| 3 | 1 | 1 | 16350.86 |
| -0.5 | 0.5 | 0.5 | 16353.32 |
| 0.5 | 0.5 | -0.5 | 16353.32 |
| 0.5 | 2 | 2 | 16358.65 |
| 2 | 2 | 0.5 | 16358.65 |
| 0.5 | 1 | 3 | 16361.25 |
| 0.5 | 3 | 1 | 16361.25 |
| 1 | 0.5 | 3 | 16361.25 |
| 1 | 3 | 0.5 | 16361.25 |
| 3 | 0.5 | 1 | 16361.25 |
| 3 | 1 | 0.5 | 16361.25 |
| -0.5 | 0 | 1 | 16366.32 |
| -0.5 | 1 | 0 | 16366.32 |
| 0 | -0.5 | 1 | 16366.32 |
| 0 | 1 | -0.5 | 16366.32 |
| 1 | -0.5 | 0 | 16366.32 |
| 1 | 0 | -0.5 | 16366.32 |
| -1 | 0 | 2 | 16366.95 |
| -1 | 2 | 0 | 16366.95 |
| 0 | -1 | 2 | 16366.95 |
| 0 | 2 | -1 | 16366.95 |
| 2 | -1 | 0 | 16366.95 |
| 2 | 0 | -1 | 16366.95 |
| 0 | 1 | 3 | 16370.24 |
| 0 | 3 | 1 | 16370.24 |
| 1 | 0 | 3 | 16370.24 |
| 1 | 3 | 0 | 16370.24 |
| 3 | 0 | 1 | 16370.24 |
| 3 | 1 | 0 | 16370.24 |
| -0.5 | -0.5 | 3 | 16370.95 |
| 3 | -0.5 | -0.5 | 16370.95 |
| -0.5 | 1 | 3 | 16376.47 |
| -0.5 | 3 | 1 | 16376.47 |
| 1 | -0.5 | 3 | 16376.47 |
| 1 | 3 | -0.5 | 16376.47 |
| 3 | -0.5 | 1 | 16376.47 |
| 3 | 1 | -0.5 | 16376.47 |
| 1 | 3 |  | 16379.16 |
| 3 | 1 |  | 16379.16 |
| -1 | 1 | 3 | 16379.73 |
| -1 | 3 | 1 | 16379.73 |
| 1 | -1 | 3 | 16379.73 |
| 1 | 3 | -1 | 16379.73 |
| 3 | -1 | 1 | 16379.73 |
| 3 | 1 | -1 | 16379.73 |
| -2 | 1 | 3 | 16381.16 |
| -2 | 3 | 1 | 16381.16 |
| 1 | -2 | 3 | 16381.16 |
| 1 | 3 | -2 | 16381.16 |
| 3 | -2 | 1 | 16381.16 |
| 3 | 1 | -2 | 16381.16 |
| -2 | -2 | -1 | 16383.06 |
| -1 | -2 | -2 | 16383.06 |
| 0.5 | 2 | 3 | 16385.34 |
| 0.5 | 3 | 2 | 16385.34 |
| 2 | 0.5 | 3 | 16385.34 |
| 2 | 3 | 0.5 | 16385.34 |
| 3 | 0.5 | 2 | 16385.34 |
| 3 | 2 | 0.5 | 16385.34 |
| -1 | 0.5 | 0.5 | 16386.52 |
| 0.5 | 0.5 | -1 | 16386.52 |
| 0 | 2 | 2 | 16390.45 |
| 2 | 2 | 0 | 16390.45 |
| -0.5 | -0.5 | 2 | 16391.89 |
| 2 | -0.5 | -0.5 | 16391.89 |
| -2 | 0.5 | 1 | 16392.37 |
| -2 | 1 | 0.5 | 16392.37 |
| 0.5 | -2 | 1 | 16392.37 |
| 0.5 | 1 | -2 | 16392.37 |
| 1 | -2 | 0.5 | 16392.37 |
| 1 | 0.5 | -2 | 16392.37 |
| -0.5 | 0 | 0.5 | 16395.78 |
| -0.5 | 0.5 | 0 | 16395.78 |
| 0 | -0.5 | 0.5 | 16395.78 |
| 0 | 0.5 | -0.5 | 16395.78 |
| 0.5 | -0.5 | 0 | 16395.78 |
| 0.5 | 0 | -0.5 | 16395.78 |
| 0.5 | 3 | 3 | 16399.66 |
| 3 | 3 | 0.5 | 16399.66 |
| -1 | 0 | 1 | 16409.73 |
| -1 | 1 | 0 | 16409.73 |
| 0 | -1 | 1 | 16409.73 |
| 0 | 1 | -1 | 16409.73 |
| 1 | -1 | 0 | 16409.73 |
| 1 | 0 | -1 | 16409.73 |
| 0.5 | 3 |  | 16413.54 |
| 3 | 0.5 |  | 16413.54 |
| -2 | -1 | -1 | 16416.41 |
| -1 | -1 | -2 | 16416.41 |
| -2 | 0 | 3 | 16419.98 |
| -2 | 3 | 0 | 16419.98 |
| 0 | -2 | 3 | 16419.98 |
| 0 | 3 | -2 | 16419.98 |
| 3 | -2 | 0 | 16419.98 |
| 3 | 0 | -2 | 16419.98 |
| -0.5 | 2 | 2 | 16422.55 |
| 2 | 2 | -0.5 | 16422.55 |
| -0.5 | -0.5 | 1 | 16425.84 |
| 1 | -0.5 | -0.5 | 16425.84 |
| -2 | -2 | -0.5 | 16428.52 |
| -0.5 | -2 | -2 | 16428.52 |
| 1 | 1 |  | 16432.86 |
| -1 | 0 | 0.5 | 16438.07 |
| -1 | 0.5 | 0 | 16438.07 |
| 0 | -1 | 0.5 | 16438.07 |
| 0 | 0.5 | -1 | 16438.07 |
| 0.5 | -1 | 0 | 16438.07 |
| 0.5 | 0 | -1 | 16438.07 |
| -2 | 0.5 | 0.5 | 16444.77 |
| 0.5 | 0.5 | -2 | 16444.77 |
| -0.5 | -0.5 | 0.5 | 16445.54 |
| 0.5 | -0.5 | -0.5 | 16445.54 |
| -2 | 0 | 2 | 16447.39 |
| -2 | 2 | 0 | 16447.39 |
| 0 | -2 | 2 | 16447.39 |
| 0 | 2 | -2 | 16447.39 |
| 2 | -2 | 0 | 16447.39 |
| 2 | 0 | -2 | 16447.39 |
| -1 | 2 | 2 | 16449.44 |
| 2 | 2 | -1 | 16449.44 |
| 0 | 2 | 3 | 16461.77 |
| 0 | 3 | 2 | 16461.77 |
| 2 | 0 | 3 | 16461.77 |
| 2 | 3 | 0 | 16461.77 |
| 3 | 0 | 2 | 16461.77 |
| 3 | 2 | 0 | 16461.77 |
| -1 | -0.5 | 3 | 16461.88 |
| -1 | 3 | -0.5 | 16461.88 |
| -0.5 | -1 | 3 | 16461.88 |
| -0.5 | 3 | -1 | 16461.88 |
| 3 | -1 | -0.5 | 16461.88 |
| 3 | -0.5 | -1 | 16461.88 |
| 0.5 | 2 |  | 16463.88 |
| 2 | 0.5 |  | 16463.88 |
| -0.5 | -0.5 | 0 | 16464.31 |
| 0 | -0.5 | -0.5 | 16464.31 |
| -2 | -1 | -0.5 | 16470.02 |
| -2 | -0.5 | -1 | 16470.02 |
| -1 | -2 | -0.5 | 16470.02 |
| -1 | -0.5 | -2 | 16470.02 |
| -0.5 | -2 | -1 | 16470.02 |
| -0.5 | -1 | -2 | 16470.02 |
| -1 | -0.5 | 2 | 16476.88 |
| -1 | 2 | -0.5 | 16476.88 |
| -0.5 | -1 | 2 | 16476.88 |
| -0.5 | 2 | -1 | 16476.88 |
| 2 | -1 | -0.5 | 16476.88 |
| 2 | -0.5 | -1 | 16476.88 |
| -2 | 2 | 2 | 16480.96 |
| 2 | 2 | -2 | 16480.96 |
| -1 | -1 | -0.5 | 16484.57 |
| -0.5 | -1 | -1 | 16484.57 |
| -2 | 0 | 1 | 16487.02 |
| -2 | 1 | 0 | 16487.02 |
| 0 | -2 | 1 | 16487.02 |
| 0 | 1 | -2 | 16487.02 |
| 1 | -2 | 0 | 16487.02 |
| 1 | 0 | -2 | 16487.02 |
| -1 | -0.5 | -0.5 | 16491.29 |
| -0.5 | -0.5 | -1 | 16491.29 |
| -1 | -0.5 | 1 | 16491.33 |
| -1 | 1 | -0.5 | 16491.33 |
| -0.5 | -1 | 1 | 16491.33 |
| -0.5 | 1 | -1 | 16491.33 |
| 1 | -1 | -0.5 | 16491.33 |
| 1 | -0.5 | -1 | 16491.33 |
| -1 | -0.5 |  | 16493.76 |
| -0.5 | -1 |  | 16493.76 |
| -1 | -0.5 | 0.5 | 16495.23 |
| -1 | 0.5 | -0.5 | 16495.23 |
| -0.5 | -1 | 0.5 | 16495.23 |
| -0.5 | 0.5 | -1 | 16495.23 |
| 0.5 | -1 | -0.5 | 16495.23 |
| 0.5 | -0.5 | -1 | 16495.23 |
| -1 | -0.5 | 0 | 16495.25 |
| -1 | 0 | -0.5 | 16495.25 |
| -0.5 | -1 | 0 | 16495.25 |
| -0.5 | 0 | -1 | 16495.25 |
| 0 | -1 | -0.5 | 16495.25 |
| 0 | -0.5 | -1 | 16495.25 |
| -2 | -0.5 | -0.5 | 16502.58 |
| -0.5 | -0.5 | -2 | 16502.58 |
| -0.5 | -0.5 |  | 16504.59 |
| -2 | 0 | 0.5 | 16507.87 |
| -2 | 0.5 | 0 | 16507.87 |
| 0 | -2 | 0.5 | 16507.87 |
| 0 | 0.5 | -2 | 16507.87 |
| 0.5 | -2 | 0 | 16507.87 |
| 0.5 | 0 | -2 | 16507.87 |
| 2 | 2 |  | 16514.95 |
| -1 | -1 | 0 | 16517.86 |
| 0 | -1 | -1 | 16517.86 |
| -2 | -0.5 | 0 | 16538.88 |
| -2 | 0 | -0.5 | 16538.88 |
| -0.5 | -2 | 0 | 16538.88 |
| -0.5 | 0 | -2 | 16538.88 |
| 0 | -2 | -0.5 | 16538.88 |
| 0 | -0.5 | -2 | 16538.88 |
| 0 | 3 | 3 | 16540.51 |
| 3 | 3 | 0 | 16540.51 |
| -2 | -1 | 0 | 16546.13 |
| -2 | 0 | -1 | 16546.13 |
| -1 | -2 | 0 | 16546.13 |
| -1 | 0 | -2 | 16546.13 |
| 0 | -2 | -1 | 16546.13 |
| 0 | -1 | -2 | 16546.13 |
| -2 | 0 |  | 16547.01 |
| 0 | -2 |  | 16547.01 |
| -0.5 | 2 | 3 | 16548.19 |
| -0.5 | 3 | 2 | 16548.19 |
| 2 | -0.5 | 3 | 16548.19 |
| 2 | 3 | -0.5 | 16548.19 |
| 3 | -0.5 | 2 | 16548.19 |
| 3 | 2 | -0.5 | 16548.19 |
| -2 | -2 | 0 | 16548.74 |
| 0 | -2 | -2 | 16548.74 |
| -1 | -1 | 0.5 | 16550.15 |
| 0.5 | -1 | -1 | 16550.15 |
| -2 | -0.5 | 0.5 | 16573.68 |
| -2 | 0.5 | -0.5 | 16573.68 |
| -0.5 | -2 | 0.5 | 16573.68 |
| -0.5 | 0.5 | -2 | 16573.68 |
| 0.5 | -2 | -0.5 | 16573.68 |
| 0.5 | -0.5 | -2 | 16573.68 |
| -1 | 0 |  | 16574.54 |
| 0 | -1 |  | 16574.54 |
| -1 | -1 | 1 | 16577.72 |
| 1 | -1 | -1 | 16577.72 |
| 0.5 | 1 |  | 16580.5 |
| 1 | 0.5 |  | 16580.5 |
| -2 | -0.5 | 1 | 16602.83 |
| -2 | 1 | -0.5 | 16602.83 |
| -0.5 | -2 | 1 | 16602.83 |
| -0.5 | 1 | -2 | 16602.83 |
| 1 | -2 | -0.5 | 16602.83 |
| 1 | -0.5 | -2 | 16602.83 |
| -1 | -1 | 2 | 16613.93 |
| 2 | -1 | -1 | 16613.93 |
| -0.5 | 0 |  | 16621.38 |
| 0 | -0.5 |  | 16621.38 |
| -1 | 2 | 3 | 16625.88 |
| -1 | 3 | 2 | 16625.88 |
| 2 | -1 | 3 | 16625.88 |
| 2 | 3 | -1 | 16625.88 |
| 3 | -1 | 2 | 16625.88 |
| 3 | 2 | -1 | 16625.88 |
| -1 | -1 | 3 | 16630.36 |
| 3 | -1 | -1 | 16630.36 |
| -2 | -1 | 0.5 | 16633.68 |
| -2 | 0.5 | -1 | 16633.68 |
| -1 | -2 | 0.5 | 16633.68 |
| -1 | 0.5 | -2 | 16633.68 |
| 0.5 | -2 | -1 | 16633.68 |
| 0.5 | -1 | -2 | 16633.68 |
| -1 | -1 |  | 16636.29 |
| -2 | -0.5 | 2 | 16639.76 |
| -2 | 2 | -0.5 | 16639.76 |
| -0.5 | -2 | 2 | 16639.76 |
| -0.5 | 2 | -2 | 16639.76 |
| 2 | -2 | -0.5 | 16639.76 |
| 2 | -0.5 | -2 | 16639.76 |
| -2 | -0.5 | 3 | 16655.29 |
| -2 | 3 | -0.5 | 16655.29 |
| -0.5 | -2 | 3 | 16655.29 |
| -0.5 | 3 | -2 | 16655.29 |
| 3 | -2 | -0.5 | 16655.29 |
| 3 | -0.5 | -2 | 16655.29 |
| -2 | -0.5 |  | 16658.98 |
| -0.5 | -2 |  | 16658.98 |
| 0.5 | 0.5 |  | 16661.22 |
| -0.5 | 3 | 3 | 16714.91 |
| 3 | 3 | -0.5 | 16714.91 |
| -2 | -2 | 0.5 | 16719.15 |
| 0.5 | -2 | -2 | 16719.15 |
| -2 | -1 | 1 | 16720.04 |
| -2 | 1 | -1 | 16720.04 |
| -1 | -2 | 1 | 16720.04 |
| -1 | 1 | -2 | 16720.04 |
| 1 | -2 | -1 | 16720.04 |
| 1 | -1 | -2 | 16720.04 |
| -2 | 2 | 3 | 16721.36 |
| -2 | 3 | 2 | 16721.36 |
| 2 | -2 | 3 | 16721.36 |
| 2 | 3 | -2 | 16721.36 |
| 3 | -2 | 2 | 16721.36 |
| 3 | 2 | -2 | 16721.36 |
| 0 | 0.5 |  | 16742.33 |
| 0.5 | 0 |  | 16742.33 |
| 0 | 1 |  | 16784.75 |
| 1 | 0 |  | 16784.75 |
| -0.5 | 0.5 |  | 16806.9 |
| 0.5 | -0.5 |  | 16806.9 |
| 0 | 2 |  | 16820.69 |
| 2 | 0 |  | 16820.69 |
| 0 | 0 |  | 16822.04 |
| 0 |  |  | 16822.04 |
| 0 | 3 |  | 16822.98 |
| 3 | 0 |  | 16822.98 |
| 2 | 3 |  | 16838.53 |
| 3 | 2 |  | 16838.53 |
| -1 | 0.5 |  | 16846.1 |
| 0.5 | -1 |  | 16846.1 |
| -2 | -1 | 2 | 16859.96 |
| -2 | 2 | -1 | 16859.96 |
| -1 | -2 | 2 | 16859.96 |
| -1 | 2 | -2 | 16859.96 |
| 2 | -2 | -1 | 16859.96 |
| 2 | -1 | -2 | 16859.96 |
| -2 | 0.5 |  | 16871.37 |
| 0.5 | -2 |  | 16871.37 |
| 0.5 |  |  | 16872.31 |
| -1 | 3 | 3 | 16876.53 |
| 3 | 3 | -1 | 16876.53 |
| -2 | -2 | 1 | 16903.72 |
| 1 | -2 | -2 | 16903.72 |
| -2 | -1 | 3 | 16949.97 |
| -2 | 3 | -1 | 16949.97 |
| -1 | -2 | 3 | 16949.97 |
| -1 | 3 | -2 | 16949.97 |
| 3 | -2 | -1 | 16949.97 |
| 3 | -1 | -2 | 16949.97 |
| -0.5 | 1 |  | 16999.78 |
| 1 | -0.5 |  | 16999.78 |
| -2 | 3 | 3 | 17073.3 |
| 3 | 3 | -2 | 17073.3 |
| -2 | -1 |  | 17126.16 |
| -1 | -2 |  | 17126.16 |
| -1 | 1 |  | 17173.6 |
| 1 | -1 |  | 17173.6 |
| -2 | -2 | 2 | 17222.47 |
| 2 | -2 | -2 | 17222.47 |
| -0.5 | 2 |  | 17291.67 |
| 2 | -0.5 |  | 17291.67 |
| 3 | 3 |  | 17305.34 |
| -2 | 1 |  | 17353.55 |
| 1 | -2 |  | 17353.55 |
| -2 | -2 | 3 | 17439.77 |
| 3 | -2 | -2 | 17439.77 |
| -0.5 | 3 |  | 17459.41 |
| 3 | -0.5 |  | 17459.41 |
| 1 |  |  | 17502.32 |
| -1 | 2 |  | 17699.81 |
| 2 | -1 |  | 17699.81 |
| -0.5 |  |  | 17765.54 |
| -2 | -2 |  | 18013.91 |
| -1 | 3 |  | 18016.06 |
| 3 | -1 |  | 18016.06 |
| -2 | 2 |  | 18124.84 |
| 2 | -2 |  | 18124.84 |
| 2 |  |  | 18498.63 |
| -2 | 3 |  | 18569.81 |
| 3 | -2 |  | 18569.81 |
| -1 |  |  | 18747.19 |
| 3 |  |  | 19018.92 |
| -2 |  |  | 19603.64 |
